# Viewing Cortical Collecting Duct Function Through Phenotype-guided Single-Tubule Proteomics

**DOI:** 10.1093/function/zqaa007

**Published:** 2020-07-02

**Authors:** Nina Himmerkus, Samuel L Svendsen, Catarina Quintanova, Markus Bleich, Otto Von Schwerdtner, Thomas Benzing, Paul A Welling, Jens Leipziger, Markus M Rinschen

**Affiliations:** 1 Institute of Physiology, Christian Albrechts University Kiel, Kiel, Germany; 2 Department of Biomedicine, Aarhus University, Aarhus, Denmark; 3 Center for Molecular Medicine, University of Cologne, Cologne, Germany; 4 Department II of Internal Medicine, University of Cologne, Cologne, Germany; 5 Departments of Physiology and Medicine, Johns Hopkins University, Baltimore, MD, USA; 6 Aarhus Institute of Advanced Studies, Aarhus University, Aarhus, Denmark; 7 Scripps Center for Metabolomics, Scripps Research, San Diego, CA, USA; 8 III. Department of Medicine, University Medical Center Hamburg-Eppendorf, Hamburg, Germany

**Keywords:** salt- and water transport, principal cell, intercalated cell, proteomics, pendrin, heterogeneity, plasticity

## Abstract

The revolution of the omics technologies has enabled profiling of the molecules of any sample. However, the heterogeneity of the kidney with highly specialized nephron segments like the cortical collecting duct (CCD) poses a challenge regarding integration of omics data and functional analysis. We examined function and proteome from the same single CCDs of C57Bl6 mice by investigating them in a double-barreled perfusion system before targeted mass spectrometry. Transepithelial voltage (V_te_), transepithelial resistance, as well as amiloride-sensitive voltage (ΔV_te_amil) were recorded. CCDs were of 400–600 µm of length, showed lumen negative V_te_ between −8.5 and −32.5 mV and an equivalent short circuit current I’_sc_ between 54 and 192 µA/cm^2^. On a single-tubule proteome level, intercalated cell (IC) markers strongly correlated with other intercalated cell markers and negatively with principal cell markers. Integration of proteome data with phenotype data revealed that tubular length correlated with actin and Na^+^-K^+^-ATPase expression. ΔV_te_(amil) reflected the expression level of the β-subunit of the epithelial sodium channel. Intriguingly, ΔV_te_(amil) correlated inversely with the water channel AQP2 and the negative regulator protein NEDD4L (NEDD4-2). In pendrin knockout (KO) mice, the CCD proteome was accompanied by strong downregulation of other IC markers like CLCNKB, BSND (Barttin), and VAA (vH^+^-ATPase), a configuration that may contribute to the salt-losing phenotype of Pendred syndrome. Proteins normally coexpressed with pendrin were decreased in pendrin KO CCDs. In conclusion, we show that functional proteomics on a single nephron segment scale allows function–proteome correlations, and may potentially help predicting function from omics data.

## Introduction

The kidney filters, reabsorbs, and metabolizes the body’s plasma volume several times a day. Water and electrolyte transport along the different nephron segments is perturbed in a variety of diseases, and can be causative for high blood pressure.^1^^,^^2^ The collecting duct is the last part of the tubular system, collecting the luminal fluid of several nephrons and determining the final urinary composition by tightly regulated secretion and reabsorption.^3–5^ The cortical collecting duct (CCD) is composed of at least three different distinct cell types that are involved in the fine-tuning of water, Na^+^, Cl^−^, K^+^, as well as proton and bicarbonate excretion.^3^^,^^5^^,^^6^

In a simplified view, principal cells reabsorb water via aquaporin-2 (apical) and aquaporin-3 and 4 (basolateral) and Na^+^ via the epithelial sodium channel (ENaC). K^+^ secretion through Renal Outer Medullary Potassium (K) channel (ROMK) is driven by the lumen-negative potential created by ENaC. Type A intercalated cells secrete protons via an apical vH^+^-ATPase. Type B intercalated cells secrete bicarbonate and reabsorb Cl^−^ via the apical Cl^−^-${\text{HCO}}_{3}^{-}$ exchanger pendrin. The chloride channel CLCNKB and its subunit Barttin (BSND) are localized at the basolateral side of intercalated cells.^7^

Considerable plasticity has been shown on a transcript level for CCD by recent single-cell transcriptomics studies, suggesting the presence of putatively “novel” cell types (or states) that might harbor determinants of biology and be very relevant for pathophysiological processes such as acidosis in chronic kidney disease.^8^^,^^9^ Compared to RNA, analysis of low-abundant proteins on a proteomics level has been more challenging because of analytical properties of proteins. Very recently, we introduced novel sample preparation methods combined with sensitive high-resolution mass spectrometers to allow proteomics interrogation of one microdissected glomerulus or one microdissected tubule at a time.^10^ This approach preserves—as compared to single-cell approaches—the original organization of the functional units of the nephron.

Here, we applied our previously developed single-tubule proteomics^11^ approach to single CCDs which had been phenotyped beforehand using perfusion and electrophysiological measurements. This approach revealed that individual CCDs showed high heterogeneity in proteome as well as in key functional properties, but correlations in between. In the pendrin knockout (KO) mouse model, the small-scale approach revealed additional information completing the functional picture of phenotype–proteome correlations. Taken together, these results functionally benchmark proteomics results and pave the way to predicting nephron function out of proteomics datasets.

## Material and Methods

### Animals

All experiments were performed in accordance with either the German law on animal protection and approved by the local authorities (animal ethics protocol number V312-72241.121-2) or in accordance with the Danish animal welfare regulations (animal experiment permission 2017-15-0202). All animals were housed under a 12-h light cycle at room temperature and standard humidity. We used animals of both sexes and in balanced sex distribution for all experiments. For functional profiling of CCDs and single isolated proteomics analysis, we used 7- to 9-week-old C57Bl6J mice. Pendrin KO mice were purchased from Jackson laboratory (JAX stock #018424) and bred in-house. The mice were kept in a pathogen-free environment. The generation of the pendrin KO mouse has been described previously.^12^ All experiments were performed with male and female mice in a C57Bl6J (Bl6) background.

### Enzymatic Tubule Preparation

Kidneys from Bl6 mice were exposed to enzymatical digestion as described in Stoessel et al.^13^ Briefly, thin coronary kidney slices were digested at 37°C and 850 rounds per minute in a thermomixer (2 mg/mL collagenase II, PAN biotechnology) in incubation solution (in mM: 140 NaCl, 0.4 KH_2_PO_4_, 1.6 K_2_HPO_4_, 1 MgSO_4_, 10 Na-acetate, 1 α-ketoglutarate, 1.3 Ca-gluconate, 5 glycine, containing 48 mg/L trypsin inhibitor, and 25 mg/L DNase I at pH 7.4). After extensive washing in incubation solution, 12 or 90 collecting ducts were collected and transferred to 5% SDS.

### Tubule Perfusion and Electrophysiology

Bl6 mice were killed by cervical dislocation and the kidneys were removed immediately. CCDs were manually dissected from kidney slices. Using a double-barreled perfusion system, trans- and paracellular electrophysiological parameters were measured at 37°C as described in Plain et al.^14^ Briefly, under symmetric conditions with luminal and basolateral physiological control solution, (in mM: 145 NaCl, 0.4 KH_2_PO_4_, 1.6 K_2_HPO_4_, 1 MgCl_2_, 1.3 calcium gluconate, 5 glucose, pH7.4) transepithelial voltage (V_te_) was recorded. Voltage deflection generated by a constant current pulse injection of 13 nA was used to estimate the transepithelial resistance (R_te_) using the cable equation. The equivalent short circuit current I’_sc_ was calculated using Ohm’s law.^15^ After an equilibration period of ∼5 min, 50 µM amiloride and 100 µM hydrochlorothiazide were applied luminally to inhibit transcellular Na^+^ transport. The peritubular bath solution was then changed for 3–5 min to a low NaCl solution (in mM: 30 NaCl, 230 mannitol, 0.4 KH_2_PO_4_, 1.6 K_2_HPO_4_, 1 MgCl_2_, 1.3 calcium gluconate, 5 glucose, pH7.4) for the generation of a NaCl diffusion potential and the calculation of the paracellular sodium chloride permeability ratio (P_Na_/P_Cl_).^14^ After returning to control solution on both sides, tubules were recovered from the bath and washed with incubation solution under visual control. Tubule diameter was measured using cable analysis. Then, they were transferred to 5% SDS and further processed for mass spectrometry as described below.

### Tubule Dissection of the Pendrin Mice

Four- to eight-week-old pendrin KO mice and their respective WT littermates (seven females and four males) were killed by cervical dislocation. Their kidneys were subsequently removed and cut in coronal slices. Five CCDs per mouse were manually dissected following the medullary rays toward the cortex. Tubules were washed in incubation solution (see above) and transferred to 5% SDS.

### Sample Processing and Mass Spectrometry Analysis

In tubular samples, proteins were denaturized by boiling at 95°C for 5 min. DTT (5 mM) and Benzonase (25 U/µL) were added and incubated for 30 min at 37°C. 2-Iodoacetamide (10 mM) was used to alkylate proteins for 45 min at room temperature. Samples were stored on dry ice and at –80°C until further processing.

The samples were thawed on ice and directly submitted to a modified, previously described SP3 protocol^11^^,^^16^ in which tryptic digestion was performed. Tubule peptides were analyzed on an nLC-MS/MS system with an easy nano-LC (Thermo scientific) coupled with a tandem mass spectrometer, a quadrupole orbitrap hybride QExactive Plus (Thermo Scientific). A 1H LC gradient on a self-packed C18 column and nanoflow (200 nL/min) was used as previously described.^17^ Raw data were searched with Andromeda embedded in the MaxQuant Version suite 1.5.3.8^18^ using the default settings against the mouse UniProt reference database released in January 2017. MaxQuant label-free quantification (LFQ) ^19^ was used for quantification. We also calculated intensity-based absolute quantification (iBAQ) values.^20^ Fixed modification was Carbamidomethyl (C), and variable modifications were Oxidation (M), Acetyl (Protein N-term). Decoy mode was revert and contaminants were included. MS precursor tolerance was 2.5 ppm. MS/MS tolerance was 20 ppm as in default settings. Deisotoping was performed. Minimum score for modified peptide was 40, and for unmodified peptides 0. PSM, Protein and Site FDR were all set to 0.01. Match between run feature was enabled, with Matching time window set to 0.7 min and Alignment time window set to 20 min (all default values). The exact parameters are also in the raw data submission.

### Untargeted Proteomics Data Analysis of Pendrin KO Mice

Andromeda-generated LFQ values were analyzed using Perseus v 1.5.5.3.^21^ Contaminant proteins were not removed to account for potential contaminations. Summed protein expression (LFQ) values were log-transformed, and normalized by subtraction of the mean. In total, 3630 proteins were discovered. Remaining missing values were imputed. A two-tailed *t*-test was performed. Proteins with fold change and P-value cutoff as indicated were used for further analysis. Data are available through ProteomExchange/PRIDE^22^^,^^23^ (see “Data Availability” section).

### Mass Spectrometry Analyses for Targeted Acquisition

Targeted mass spectrometry data were obtained using the inclusion list option as a parallel reaction monitoring approach. For a list of included masses as well as their retention times, see Supplementary Table S1. The spectral database was generated from the data-dependent spectral library of the initial CCD profiling experiments, and the data was analyzed and quantified on MS2 level using skyline^24^ as previously described.^11^ In brief, these filters required (1) a coelution of MS2, (2) a mass error <10 ppm, and (3) elution within a predefined acquisition window. Then, data were normalized by log_2_ transformation, and subtraction of the mean from both columns and rows as described in Höhne et al.^11^ All transitions were manually validated for peak shape and signal to noise and excluded if they did not meet the appropriate quality criteria.

### Immunofluorescence and Western Blot

Kidney slices from either pendrin KO or their littermates (aged 4 months, *n* = 3, 3, 4 female, 2 male) were enzymatically digested as described above. And 8–12 collecting ducts were collected and transferred to poly-lysine coated slides. CCDs were fixed with 4% PFA. Tubules were extensively washed in dissection solution, followed by washing with PBS-T (0.3% triton-X-100 in PBS). Tubules were exposed to primary antibodies in 5% BSA in PBS-T (Table 1) overnight at 4°C. After washing, tubules were incubated with secondary antibodies (1:300, Alexa Fluor 488, or −633) for 1 h at room temperature. Samples were mounted using Mowiol-Dabco containing DAPI (4',6-diamidino-2-phenylindole) for staining of nuclei. Confocal images were acquired using Zeiss LSM 880 with Airyscan (laser with wavelengths of 488 and 633 nm). Cells positive for vH^+^-ATPase, Barttin, or AE1 were counted and normalized for tubular length. In parallel, images recorded with the same settings were transformed to 8-bit and analyzed in imageJ for intensity of vH^+^-ATPase, Barttin, and AE1 staining using the same threshold for all preparations to only measure stained tubular areas. Intensity was then normalized for tubular length and is given in arbitrary units.

**Table 1.** Antibodies Used in This Study

Antibody	Catalogue Number or Paper Reference	Application	Dilution
Guinea pig anti-pendrin	Kind gift of C.Wagner, Zürich^25^	*IF*	*1:600*
		*WB*	*1:2000*
Rabbit anti-vH^+^-ATPase, B1 subunit	Kind gift of J. Praetorius, Aarhus^26^	*IF*	*1:300*
		*WB*	*1:2000*
Rabbit anti-Barttin	Kind gift of T.Jentsch, Berlin^7^	*WB*	*1:3000*
Guinea pig anti-Barttin	Kind gift of T.Jentsch, Berlin^7^	*IF*	*1:450*
Rabbit anti-GAPDH	Cell Signalling (14C10)	*WB*	*1:1000*
Alexa Fluor 488 and 633	Thermofisher (A21206, A21070, A11073)	*IF*	*1:300*
Anti rabbit-HRP	Dianova (111-035-144)	*WB*	*1:50 000*
Anti-AE1	Kind gift of C. Wagner, Zürich^27^	*IF*	*1:1000*

In parallel, batches of 30 CCDs were transferred into Laemmli-buffer and stored at −20°C. For Western blotting a sample of each mouse was heated to 95°C for 5 min, separated by SDS-PAGE and blotted onto nitrocellulose membrane. Membranes were blocked with 5% bovine serum albumin in PBS-Tween (0.1% Tween-20 in PBS). Primary antibodies (Table 1) were incubated at 4°C overnight. After extensive washing with PBS-Tween, secondary antibodies were incubated at room temperature for 1–2 h, membranes washed again, and blots developed (ChemiDoc MP, Bio-Rad Laboratories). Steps were repeated with GAPDH primary antibody (Table 1) for semiquantitative analysis. Bands were analyzed using Image Lab software (Version 5.0, Bio-Rad Laboratories) and vH^+^-ATPase and Barttin bands were normalized for the respective GAPDH bands.

### Data Availability

The data are available through PRIDE/proteomExchange consortium^22^^,^^23^ under the following identifiers and the URL https://www.ebi.ac.uk/pride/archive/login: : PXD018795, PXD018792, PXD018791.

### Statistical Analysis

The statistical analysis of mass spectrometry data is described in the respective sections. The statistical analysis of the Western blot was performed using GraphPadPrism 7.0 and *t*-test, and simple Pearson’s correlation analysis. Boxplots were redrawn with instant clue.^28^ The chord diagrams were generated using R circlize package using the following settings: grid color according to cell types, link.sort = TRUE, link.decreasing = TRUE, reduce = 0.00, SCALE = FALSE.

## Results

**Figure 1.**
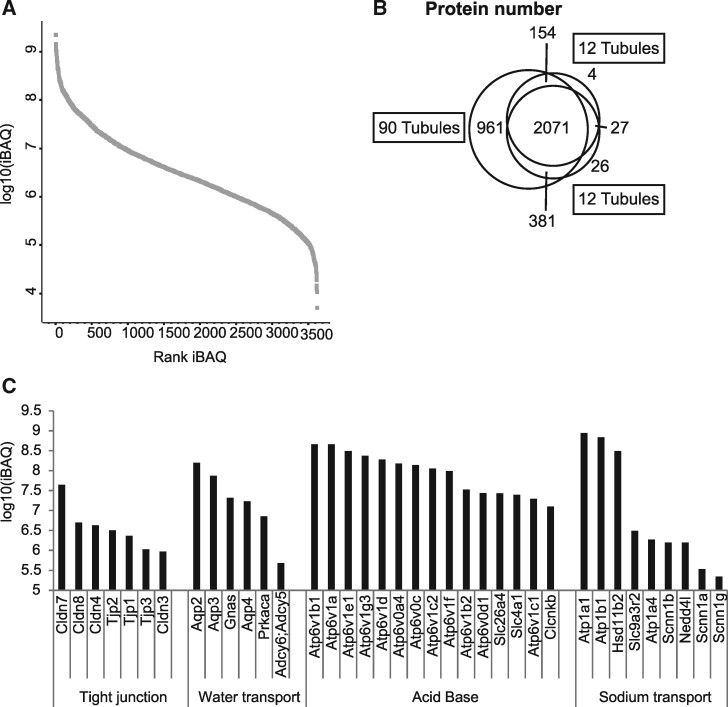
Profiling of Manually Dissected Mouse CCDs for Generation of a Spectral Library**. (A**) Dynamic range of profiling of CCD proteins. (**B)** Venn diagram of different CCD profile datasets used for spectra library generation. (**C**) Relative stoichiometry of different regulatory protein complexes and functional protein families involved in CCD function.

We analyzed isolated CCD segments using mass spectrometry-based proteomics in order to generate a spectral library of the tissue. Spectral libraries can be used for targeted and data-independent proteomic interrogations of tissues. In a first round, we investigated two sets of only 12 tubules and one with 90 CCDs pooled together. The generated proteomics profile contained around 3630 proteins. Using copy number estimation, the data show that the proteome spans 5 orders of magnitude (Figure 1A). Scaling up the preparation from 12 to 100 tubules improved resolution and spectral and protein amount (Figure 1B). The generated proteomics profile contained data from all three major cortical collecting duct cell types: principal cells (PCs); Type A and B intercalated cells (ICs) (Figure 1C; see also discussion in Figure 6B). The bar plot represents intensity-based absolute quantification values, a surrogate for protein copy numbers. Relatively high expressed proteins are the ubiquitously expressed Na^+^K^+^ ATPase subunits. Aquaporins (Aqp2, 3, 4) are proteins involved in vasopressin-stimulated water transport and typical representatives of the PC proteome. The copy number of the other main PC feature, ENaC, on the other hand, was much lower (subunits SCNN1a, SCNN1b, SCNN1c). Proteins involved in acid–base homeostasis are found in type A IC (AE1 SLC4a1) as well as in type B IC (Pendrin Slc26a4, see also Figure 6B). In addition, we show here a set of tight junction proteins with lower expression but attributed to all cells. Thereby, we have generated a spectral library that allows targeted interrogation of single isolated and phenotyped CCD segments.

**Figure 2.**
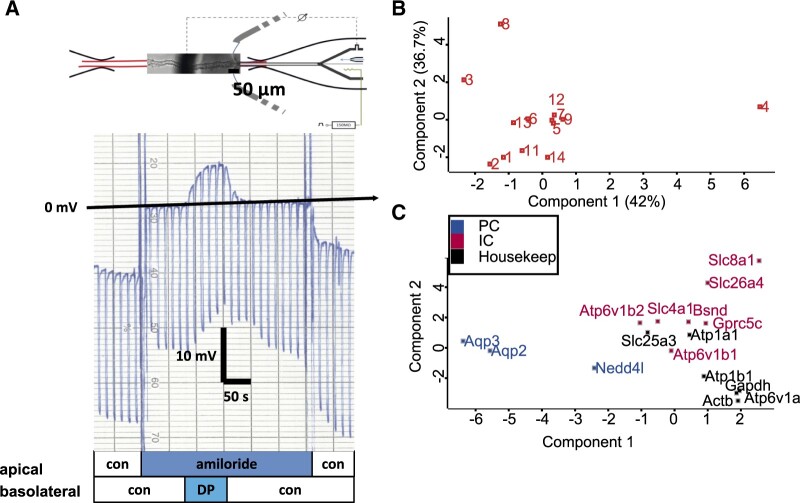
Electrophysiological Analysis of Isolated Perfused CCDs and Tubule Proteome Quality Controls. **(A)** Representative chart recording of an isolated perfused CCD. In the presence of luminal amiloride (as marked below) the negative voltage is abolished and a diffusion potential (DP) is generated (positive) by the basolateral application of dilute isotonic NaCl. (**B**) PCA of individual CCD tubules proteomes. (**C**) PCA of individual protein expressions from single tubule proteomics datasets.

We then perfused 14 CCDs using a double-barrel perfusion system and measured functional parameters (Figure 2A), out of which we recovered 12 for proteomics. The protocol involved treatment with amiloride and change of the basolateral sodium content. The parameters included morphological (such as tubule length and width) as well as electrophysiological data (such as V_te_ and resistance, see Supplementary Figure S1 and Table S2). An additional two tubules were obtained under the same conditions without perfusion. Based on the spectral library (Figure 1), we designed a targeted proteomics assay for PC markers, type A IC markers, and type B IC markers. The analysis revealed heterogeneity of the dataset (for a PCA, see Figure 2B). Performing the same analysis for individual proteins, we demonstrated distribution of different cell markers as the main driver for the first component, suggesting that the IC/PC proportion was driving the key variance in the dataset (Figure 2C). Some targeted proteins, such as claudin, had insufficient readings to include further in the analysis.

**Figure 3.**
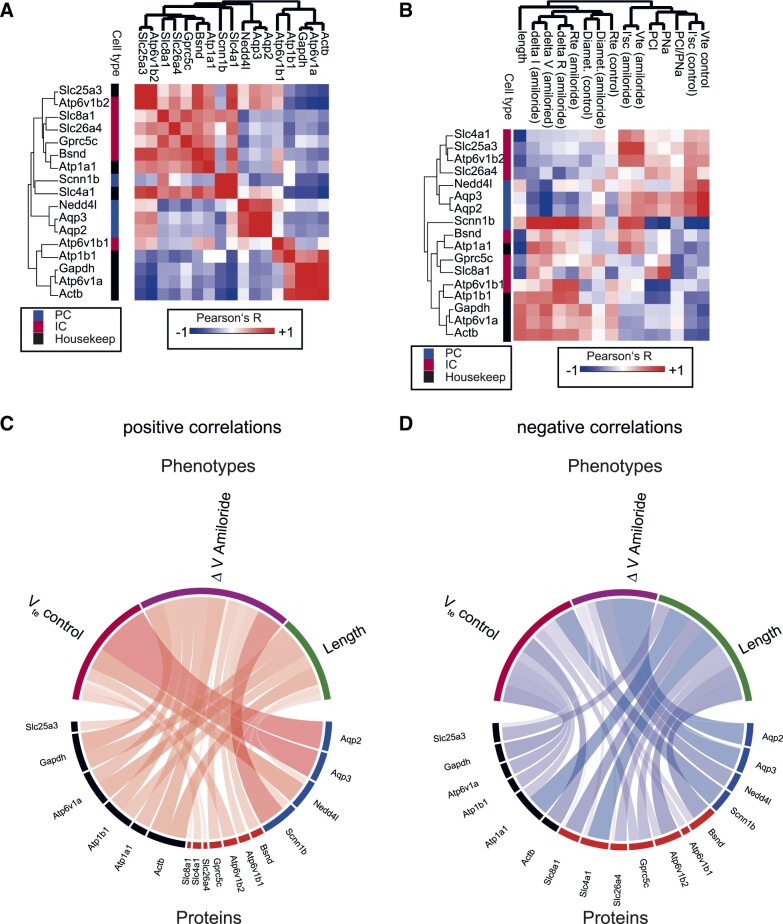
Cell–Cell and Cell–Function Relationships of Individual Perfused CCDs. (**A**) Heatmap demonstrating correlation coefficients between individual proteins. Proteins with similar cellular localization (IC, PC, and “housekeeping”) genes cluster together in hierarchical clustering (Euclidean distance). (**B**) Heatmap demonstrating correlation coefficients between individual proteins and functional and morphological parameters (Euclidean distance). (**C**) Chord diagram demonstrating relative contribution of total net positive correlation between three functional parameters and protein markers for every three cell types. (**D**) Chord diagram demonstrating relative contribution of total net negative correlation between three functional parameters and protein markers for every three cell types.

We then integrated protein expression levels as determined by peptide intensity with the phenotypic parameters measured using the classical physiological technique. Analyzing protein–protein variation, we found that intercalated cell markers shared a high correlation with each other, whereas this set of proteins had a negative correlation with PC markers (Figure 3A). We then integrated the data with electrophysiological and morphometric phenotypes. The dataset comprises a correlation between amiloride-sensitive voltage (ΔV_te_ amiloride) and one subunit of the channel responsible for this voltage, beta-ENaC (*R* = 0.78). In addition, it revealed relationships that were unexpected, such as inverse correlation between (ΔV_te_ amiloride) and AQP2 (Figure 3B). Notably, the tubule length correlated positively with the amount of actin. Integrated data visualization was performed using a chord diagram (or Circos plot) using the R circlize package. The thickness of the chords illustrates the relative contribution of individual correlation coefficients to the global correlation (Figure 3C, D). We separated and visualized positive and negative correlations between proteins and phenotypes. The data showed that ENaC was strongly correlated with the parameter of ΔV_te_ amiloride, whereas AQP2 and 3 strongly correlated positively with the parameter of basal negative V_te_ (Figure 3C). Note that the correlation between AQP2/3 and basal V_te_ is positive because the V_te_ is negative, i.e. the more the AQP2/3 expression, the closer the voltage is to zero. The main contributors to positive correlations to the morphological parameter “length” were housekeeping genes (Figure 3C). Negative correlations were observed between ΔV_te_ amiloride and aquaporins (Figure 3D). The main contributor to the morphological parameter “length” (negative correlation) were proteins expressed in intercalated cells—the longer the tubule, the less intercalated cell protein markers (Figure 3D).

**Figure 4.**
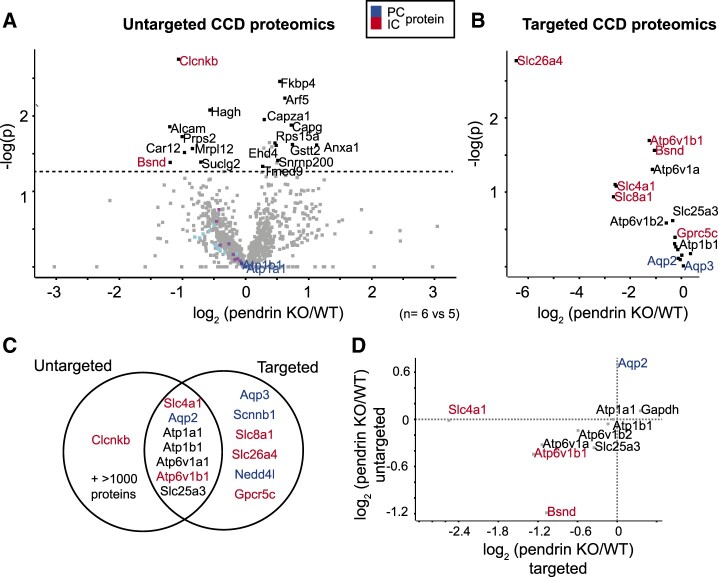
Untargeted and Targeted CCD of a Well-Characterized Mouse Model Reveals Novel Mechanisms in the Pendrin KO. (**A**) Volcano plot quantification of untargeted proteomics data of CCDs isolated from pendrin KO versus control littermates. (**B**) Volcano plot quantification of targeted proteomics data of CCDs isolated from pendrin-KO versus control littermates (**C**) Venn diagram comparing functionally relevant transport proteins identified using targeted or untargeted proteomics data. Untargeted protoemics acquisition covered different proteins as targeted acquisition and let to the discovery of CLCNKB. (**D**) Scatterplot comparing log_2_ ratios in untargeted and targeted proteomics dataset.

Since protein expression appeared to vary according to cell-type composition, we asked whether changes in proteins in distinct cell types would lead to a change in other, functionally coupled proteins. To this end, we analyzed CCDs of pendrin KO mouse, a mouse model that mimics the phenotype of the human Pendred syndrome (OMIM: 274600).^29^ First, we performed an untargeted analysis of isolated CCDs from the KO mouse in comparison to their respective control littermates (Supplementary Table S3). A strong decrease of the CLCNKB channel was observed, combined with a decrease of BSND, the gene causative in type 4 Bartter syndrome (Barttin, OMIM: 602522) (Figure 4A).^30^ Also, several subunits of the vH^+^-ATPase (Figure 4A, cyan dots) were found downregulated in pendrin KO CCD. In addition to downregulation of these marker proteins, the A-cell specific marker Slc4a1 (AE1) was not changed. Additional targeted analysis revealed a very strong decrease of pendrin, as expected (Figure 4B, protein abundance in KO is noise signal, Supplementary Table S4). Interestingly, analysis confirmed a decrease of BSND, the subunit of the chloride channel CLCNKB. Several other proteins were also changed (Figure 4C, Supplementary Table S4), including the IC-specific vH^+^-ATPase B subunit. ROMK was covered by neither of these approaches, and ENaC amount (beta-subunit) was not changed. Expectedly, untargeted and targeted analyses were complementary (Figure 4C), but overall consistent in the degree of changes (Figure 4D).

**Figure 5.**
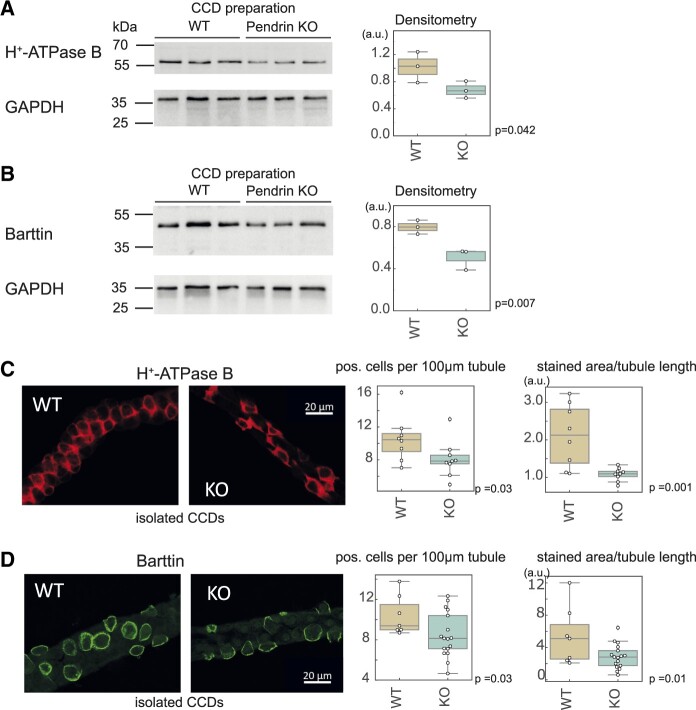
Confirmation of Targeted and Untargeted Proteomics Analysis using Western Blot and Immunofluorescence. Immunoblot and densitometry of vH^+^-ATPase (B1-subunit) (**A**), and Barttin (**B**). Immunoblot from 30 tubules from *n* = 3 mice. A significant decrease was observed, consistent with untargeted and targeted proteomics results. Immunofluorescence for vH^+^-ATPase (B1-subunit) (**C**) and for barttin (**D**) of single isolated tubules mounted on coverslips from a tubule. Each dot represents an observation from a single tubule. In total, *n* = 3 mice per group.

We sought to corroborate these findings by Western blot analysis. BSND and vH^+^-ATPase subunit B were decreased in the pendrin KO, consistent with proteomics results (Figure 5A, B). Immunofluorescence analysis of isolated microdissected tubules confirmed the changes (Figure 5C, D) with a consistently reduced number of cells positive for these proteins. As a technical validation, the unique typical expression patterns of vH^+^-ATPase B1 subunit and Barttin in microdissected nephron segments were confirmed by immunoblot, revealing strong expression of both proteins in the distal nephron (Supplementary Figure S2A, B). Pendrin KO was confirmed by immunofluorescence (Supplementary Figure S2C).

We further analyzed whether the decrease of pendrin and vH+-ATPase B1 and CLCNKB subunit was associated with a loss of intercalated cell markers. We mapped proteins whose corresponding transcripts were shown to be specifically expressed in mouse CCD in either A-IC, B-IC, both IC combined or PC^9^ (Supplementary Figure S3A, B). The analysis showed that PC markers tended to be increased and IC markers tended to be decreased, with only two marker proteins reaching significance (Carboanhydrase12, a protein immunolocalized to A-IC^31^ and suggested as a transcriptomic IC marker for both A-IC and B-IC cells^9^ was decreased, and Anxa1, a PC marker was increased). A-IC markers were not changed and only one B-IC marker (Uap1l1) was quantified on protein level that was not changed. Consistent with these results, we found that AE1 (Slc4a1) cell numbers in immunofluorescence were not significantly increased, consistent with previous literature^32^ (Supplementary Figure S3C, D).

**Figure 6.**
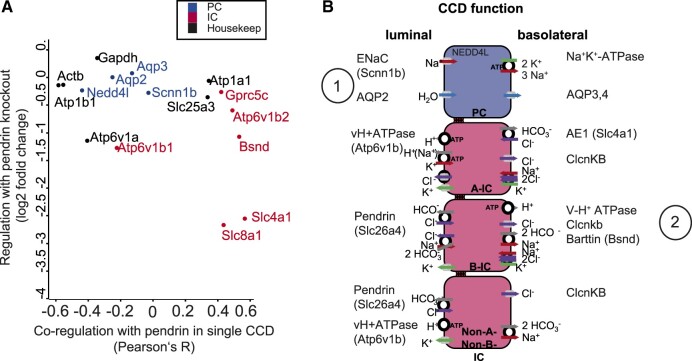
**Summary of Findings.** (**A**). Integration of tubule-to-tubule co-variation with pendrin (from Figure. 3) and regulation in the pendrin KO mouse (from Figure 4). Proteins positively associated with pendrin in normal single tubules are more likely to be decreased in the pendrin KO mouse. (**B**) Simplified schemes of PC and IC cell types in CCD. Membrane proteins and regulators (detected in this study) are depicted at their luminal and basolateral site of expression. 1 represents functional relationship between ENaC, AQP2, and NEDD4; 2 represents coregulated protein in pendrin KO CCD.

In single perfused CCD proteomics, we found that proteome heterogeneity in single collecting ducts underlies the functional heterogeneity in these tubules. From pendrin KO CCD proteomics, we learned that proteome machineries involved in cell type-specific directed transport are coupled, here in particular in the B cells. Combining both analyses regarding protein expression, we found that the proteins impaired by pendrin KO (Figures 4, 5) also have high co-expression with pendrin in “normal” (WT) single collecting ducts (Figure 3). Figure 6A shows a scatterplot revealing this connection.

## Discussion

The mission of physiology is to explain cell, tissue, and whole-organism function from biochemical and physical empirical data. With the advent of the omics, the explosion of—often semiquantitative—biochemical data has outpaced our capability to understand what these data mean for a system, or an organism.^33^ While more and more sensitive methods are used to profile molecular composition of fewer and fewer amounts of cells and tissues, the actual functional relevance of these changes (for example in single genes) is unclear, and often validated in rather crude KO models.^34^ The question about functional relevance of any omics dataset will also decide on the success of the coming omics-based approaches to “precision medicine,” especially since protein and transcript only correlate moderately.^35^

The kidney CCD is one of the most heterogenous and plastic epithelia in the human body. Here we used single-nephron functional proteomics that revealed coupling of expression signatures in a functional context. The proteomic accessibility to targeted analyses enables a better dynamic range, and a better and further increased reproducibility of signals over noise, especially for small sample amounts as compared to untargeted analyses.^11^^,^^36^ With this regard, the methods described here provide a novel window into the functional relevance of an omics dataset, thereby benchmarking this proteomics dataset. We observed that CCDs are indeed heterogenous, a fact long known and corroborated by recent single-cell sequencing studies.^8^ Correlation of function and protein expression was largely consistent with known physiological function. For instance, ENaC correlated positively with ΔV_te_ amiloride. On a cell level, high levels of ENaC were associated with low levels of Nedd-4-2. The positive correlation between AQP2 and V_te_, and the negative correlation between AQP2 and ΔV_te_ amiloride revealed a surprising heterogeneity of collecting ducts or PCs that are either more adapted for water transport (AQP2) or for Na^+^ transport (ENaC). These results suggest that even “traditional” circuits in kidney physiology can be reevaluated and expanded using molecular composition of phenotyped samples. The negative correlation between tubular length and intercalated cell markers can be explained by the decreased percentage of intercalated cells in the more medullary parts of the CCD.

Even in a well-known model, we found unanticipated expression wirings. Pendrin mediates the exchange of chloride and bicarbonate at the apical membrane of intercalated cells.^29^ Mutations in the gene coding for pendrin leads to Pendred syndrome. Patients suffering from Pendred syndrome present with sensorineural deafness, goiter and, importantly, are prone to develop severe metabolic alkalosis.^37^ The latter is elaborated by the finding that pendrin KO mice have lower urinary pH and do not respond with a urinary alkalization when challenged with an acute bicarbonate load.^38^ This is caused by a defect pendrin-dependent bicarbonate secretion from B-ICs.^29^^,^^39^ Moreover, it is well established that pendrin in concert with basolateral Cl^−^ channels mediate transcellular Cl^−^ transport.^40^ Here we report another aspect of this functional coupling, namely that pendrin KO is functionally coupled with decreases in CLCNKB and Barttin protein abundance, the channel complex mutated in Bartter syndrome (Figures 3, 4). This suggests that the salt wasting character observed in pendrin KO mice,^41^ and potentially in patients with Pendred syndrome, is connected to alterations in members of the Bartter complex (Figure 4, 6B). Based on analysis of other protein markers, and the previous literature, it seems unlikely that this phenotype is only caused by a loss of B-ICs: Only one PC marker is significantly increased, consistent with a tendency for more PCs in previous studies of pendrin KO CCD. The number of AE1-positive cells expressed in the A-IC does not change. Instead, the data are more compatible with a CCD remodeling of pendrin-related functionally related partners. The fact that interaction partners are coregulated when a main, high-abundant protein is missing was observed before in patients with defective Actinin-4,^17^ as well as nephrin mutations,^11^ thereby confirming the paradigm observed here.

The discussion of these data reveals the strengths and weaknesses of this approach. The functional and molecular discovery in one integrated dataset is a key strength. Notably, the heterogeneity is resolved on a functional unit and protein level, posing an additional level information as compared to single-cell approaches. A clear strength is the discovery approach: An ancillary finding was downregulation of carbonic anhydrase 12, a largely uncharacterized membrane carbon dioxide fixing protein whose transcript can be found in both types of ICs (Supplementary Figure 3) and has been localized strongly to A-ICs. In addition, this approach allows a new view point on tubules as it analyzes single tubules as “functional individuals,” potentially unraveling local regulation in contrast to whole organism/whole kidney or single-cell regulation. Weaknesses are the low throughput (on the level of tubule perfusion correlations) and still limited sensitivity that will improve by further generations of mass spectrometers—the proteome depth is currently in the range of several hundreds of proteins. Correlations are also never causations.

In conclusion, the unique advance combining omics, function, and phenotype in single-nephron segments enables both functional benchmarking of integrative omics datasets, as well as new insights into kidney function and physiology.

## Supplementary Material

zqaa007_Supplementary_Data
